# Contribution of local and surrounding anthropogenic emissions to a particulate matter pollution episode in Zhengzhou, Henan, China

**DOI:** 10.1038/s41598-023-35399-8

**Published:** 2023-05-30

**Authors:** Yaobin Wang, Feng Wang, Ruiqi Min, Genxin Song, Hongquan Song, Shiyan Zhai, Haoming Xia, Haopeng Zhang, Xutong Ru

**Affiliations:** 1 Key Laboratory of Geospatial Technology for the Middle and Lower Yellow River Regions, Ministry of Education, Henan University, Kaifeng, 475004 Henan China; 2 Henan Key Laboratory of Integrated Air Pollution Control and Ecological Security, Henan University, Kaifeng, 475004 Henan China; 3 Institute of Urban Big Data, College of Geography and Environmental Science, Henan University, Kaifeng, 475004 Henan China

**Keywords:** Atmospheric science, Climate change

## Abstract

In this study, we simulated the spatial and temporal processes of a particulate matter (PM) pollution episode from December 10–29, 2019, in Zhengzhou, the provincial capital of Henan, China, which has a large population and severe PM pollution. As winter is the high incidence period of particulate pollution, winter statistical data were selected from the pollutant observation stations in the study area. During this period, the highest concentrations of PM_2.5_ (atmospheric PM with a diameter of less than 2.5 µm) and PM_10_ (atmospheric PM with a diameter of less than 10 µm) peaked at 283 μg m^-3^ and 316 μg m^-3^, respectively. The contribution rates of local and surrounding regional emissions within Henan (emissions from the regions to the south, northwest, and northeast of Zhengzhou) to PM concentrations in Zhengzhou were quantitatively analyzed based on the regional Weather Research and Forecasting model coupled with Chemistry (WRF/Chem). Model evaluation showed that the WRF/Chem can accurately simulate the spatial and temporal variations in the PM concentrations in Zhengzhou. We found that the anthropogenic emissions south of Zhengzhou were the main causes of high PM concentrations during the studied episode, with contribution rates of 14.39% and 16.34% to PM_2.5_ and PM_10_, respectively. The contributions of anthropogenic emissions from Zhengzhou to the PM_2.5_ and PM_10_ concentrations in Zhengzhou were 7.94% and 7.29%, respectively. The contributions of anthropogenic emissions from the area northeast of Zhengzhou to the PM_2.5_ and PM_10_ concentrations in Zhengzhou were 7.42% and 7.18%, respectively. These two areas had similar contributions to PM pollution in Zhengzhou. The area northeast of Zhengzhou had the lowest contributions to the PM_2.5_ and PM_10_ concentrations in Zhengzhou (5.96% and 5.40%, respectively).

## Introduction

Particulate matter (PM) in the ambient air is a mixture of solid particles and liquid droplets, which are mainly generated from natural sources (e.g., dust events, wildfires, volcanic eruptions, and sea spray), anthropogenic emissions (e.g., vehicular emissions, industrial emissions, power plants, and household emissions), and atmospheric transformation^1–4^. PM includes inhalable particles with diameters that are generally less than 10 µm (PM_10_) and fine particles with diameters that are generally smaller than 2.5 µm (PM_2.5_)^5^. Inhalable particles pose great risks to human health by lodging deep into the lungs, and some may even enter the human bloodstream^6–9^. Fine particles can also reduce visibility, change the radiative balance, and affect the diversity of ecosystems^10–15^. China has experienced severe air pollution characterized by high concentrations of particulate matter due to rapid economic development, urbanization, and industrialization over the past several decades, especially in highly populated and developed urban regions such as the Pearl River Delta (PRD), the Yangtze River Delta (YRD), and the Beijing-Tianjin-Hebei (BTH)^16–20^.

Source apportionment that quantifies the contribution of sources to air pollutants is the basis for formulating air pollution control strategies and includes two methods of receptor models and air quality models^21^. Receptor models such as chemical mass balance (CMB)^22,23^ and positive matrix factorization (PMF)^23–25^ can estimate the relationship between receptors and sources on the basis of measurements. Numerous studies have been conducted to quantify the contribution of emission sources to PM in Chinese cities, especially over the regions of the PRD, YRD and BTH^26–28^. However, receptor models still show great uncertainty because they often adopt a fixed profile for secondary sources^21^ and cannot distinguish whether the contribution of local or regional transport plays a leading role in formulating PM control strategies^29^. In addition, the source apportionment results of receptor models have had limited spatial coverage due to the limited samples and large spatial span of potential sources and receptor sites^30^.

Air quality models use mathematical and numerical techniques to simulate the physical and chemical processes that affect the dispersion, formation, transport, and deposition of air pollutants in the atmosphere. They have been recognized as a useful tool for air pollution controls due to their ability and large spatial coverage to quantify the transport impacts of regional air pollutants^31–35^. Many studies have been conducted to quantify the contributions of regional sources to PM over severely polluted regions of China, such as BTH^36^, PRD^37–39^, YRD^40^, North China Plain (NCP)^41^, and western China^42,43^, by using air quality models, such as the Weather Research and Forecasting model coupled with Chemistry (WRF/Chem)^44^ and the Community Multiscale Air Quality model (CMAQ)^45^. They mostly used air quality models to simulate the concentration of particulate matter in the study area at a time when the region is prone to heavy air pollution (generally, the simulation time is half a month to one month). Therefore, they can evaluate the model performance by comparing the model results to the monitoring data and analyzing the pollutant variability and influencing meteorological factors in the study region^32,33^. Chen et al. found that the ambient PM_2.5_ at Lingcheng (a district of Dezhou city in Shandong Province) was affected not only by emissions from local and circumjacent areas; emissions resulting from regional and long-range transport also needed to be considered. Chang et al. found that in July, the local contributions to PM_2.5_ pollution in Beijing were only 33%, with contributions of approximately 3.6–5.3 μg m^-3^ coming from Shandong Province and Henan Province. These findings provide a basis for the design and implementation of emission control strategies to improve the regional air quality of China.

Most of the previous studies on PM source apportionment in China using air quality models mainly focused on populated and economically developed regions, such as the PRD, YRD, and BTH. Henan is the most populous province in central China and has become one of the most severely PM-polluted regions of China^33^. Zhengzhou, the capital of Henan Province, is located in the air pollutant transport route from the severely polluted region of BTH and suffers severe particulate matter pollution problems, especially in winter^46^. Urban PM is mainly generated from sources such as vehicle emissions, road/soil dust, biomass burning, agricultural emissions, and regional transport aerosols; however, studies have not yet quantified the contribution of each source or explained the formation mechanism of PM^5,47^. In recent years, several studies have quantified the source apportionment of PM_2.5_ in Zhengzhou by using receptor models^46,48–50^. Nevertheless, the contributions of local emissions and regional transport to PM in Zhengzhou and Henan Province remain unclear, which makes it difficult to understand the source and formation mechanism of PM in this region.

Winter is the high incidence period of particulate pollution, hence, the simulation study period was selected according to the statistical data of pollutant observation stations in the study area. A severe PM pollution event in the winter of 2019 in Zhengzhou gave us the opportunity to study the local and regional transport of PM pollution in Zhengzhou as a first step toward understanding where the pollution in Zhengzhou originates. We adopted the WRF/Chem model to quantify the contributions of local and surrounding anthropogenic emissions within Henan Province to particulate matter concentrations during a severe PM pollution episode from December 10–29, 2019, in Zhengzhou. The contribution rates of local emissions and emissions in areas northeast (Xinxiang and Kaifeng), northwest (Luoyang and Jiaozuo), and south (Pingdingshan and Xuchang) to Zhengzhou were analyzed during this pollution episode. The findings of this study may provide data and model references for subsequent relevant research and the scientific and rational guidance of local PM pollution control policy-making.

## Materials and methods

### WRF/Chem model

WRF/Chem is a collaboration between several organizations, principally the National Oceanic and Atmospheric Administration (NOAA), National Center for Atmospheric Research (NCAR), Pacific Northwest National Laboratory (PNNL) and National Aeronautics and Space Administration (NASA), as well as many other institutes^51,52^. The model is a next-generation mesoscale numerical weather prediction system designed to serve both operational forecasting and atmospheric research needs. With the temporal and spatial resolutions completely connected online with a meteorological module and chemical module, all the emissions, transport, mixing, and chemical transformations of trace gases and aerosols can be modeled simultaneously with the meteorological module^53^. Grell et al.^54^ described WRF/Chem in detail, and Tie et al.^55^ modified its chemical scheme. More detailed descriptions of WRF/Chem can be found in previous studies, such as Grell et al.^54^. The performance of the WRF/Chem model for air pollutant concentration simulation has been verified by many studies^31,51,52,56^. The map of the WRF/Chem simulation results is created in NCAR Command Language (NCL, https://www.ncl.ucar.edu/).

### Hysplit-4 model

The HYSPLIT-4 model was jointly developed by the Air Resources Laboratory of the National Oceanic and Atmospheric Administration (NOAA) and the Australian Bureau of meteorology. It can be used to calculate a simple air mass trajectory and simulate complex diffusion and sedimentation, such as sand dust, PM_2.5_, fire, volcanic ash, etc. At present, it has been widely used in the calculation of backward trajectories and the study of pollutant transport and diffusion^57^. It can qualitatively understand the potential sources of pollutants by simulating the area through which the air mass passes before reaching the area of concern.

### Model settings

**Figure 1:**
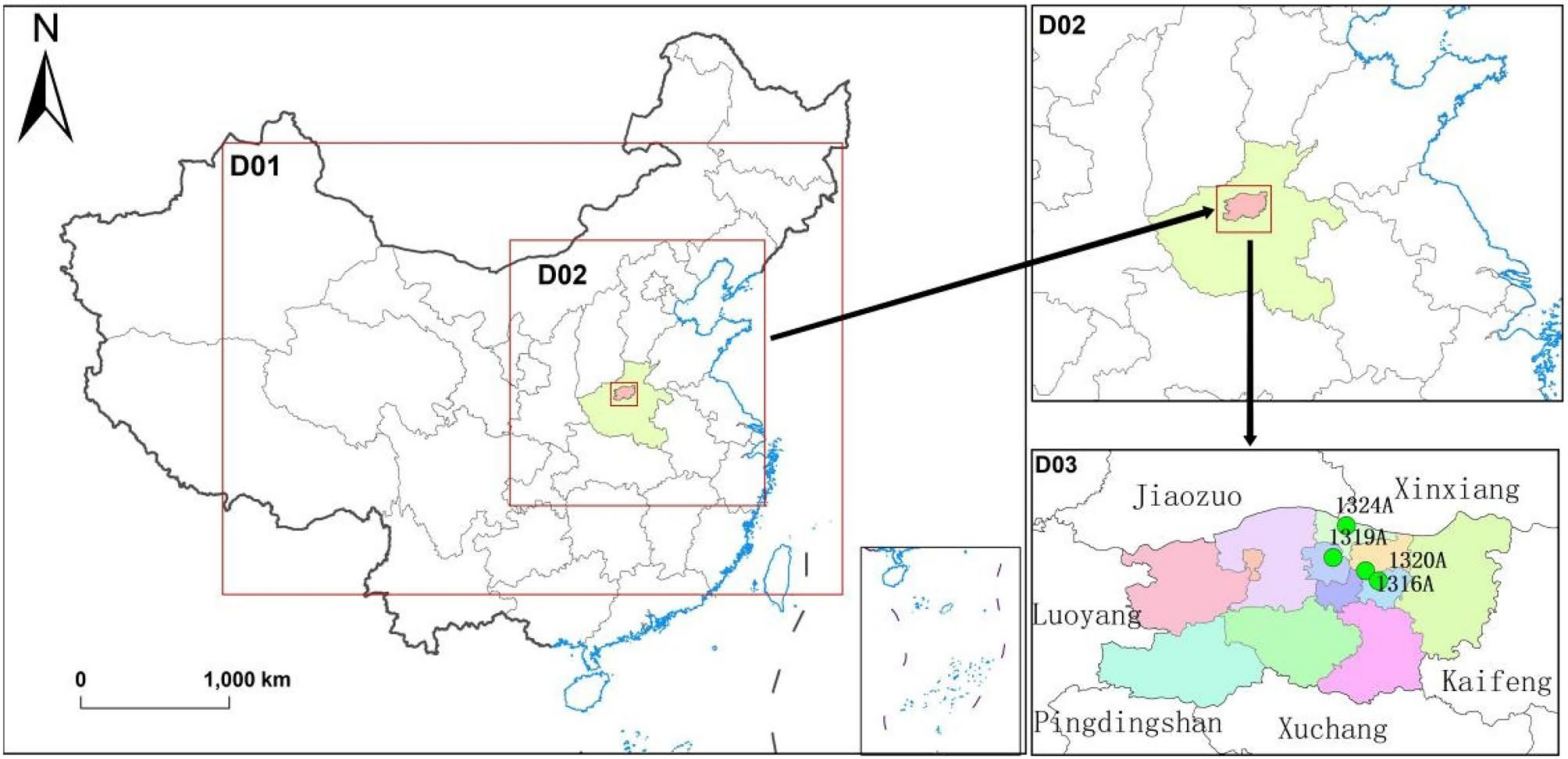
Simulation domain configuration of the WRF/Chem model.

In this study, a triple-nested region was implemented from China to Zhengzhou (Fig. 1). Domain 1 comprised a large area of China with a horizontal resolution of 27 km and mainly provided initial and boundary conditions for the inner grids. Domain 2 included central China and North China at a horizontal resolution of 9 km, and Domain 3 spanned Zhengzhou and its surrounding areas at a horizontal resolution of 3 km. A spin-up period of 168 h was used to minimize the influence of the initial conditions. The vertical structure of the model includes 34 layers covering the whole troposphere. The chemical conditions at the lateral boundaries were constrained by a global chemical transport model. The Lin et al. microphysics scheme^58^, the Mellor-Yamada-Janjic (Eta) turbulent kinetic energy (TKE) scheme, and the Noah land surface model were used in this study. The atmospheric shortwave and longwave radiation fluxes were computed using the (old) Goddard shortwave scheme and Goddard scheme, respectively. The Carbon Bond Mechanism version Z (CBMZ) model was used as the gas-phase chemistry scheme^59^. The Madronich fast tropospheric-ultraviolet visible (F-TUV) photolysis scheme was used for the particulate matter simulations. Table 1 shows the WRF/Chem configurations of the physical and chemical options.

**Table 1:** Physics and chemistry options used for the simulation cases.

Type	Scheme	Domain 1	Domain 2	Domain 3
Physics	Microphysics	Lin et al	Lin et al	Lin et al
	Planetary boundary layer	MYJ	MYJ	MYJ
	Cumulus parameterization	GF	GF	GF
	Longwave radiation	New Goddard	New Goddard	New Goddard
	Shortwave radiation	Goddard	Goddard	Goddard
	Land-surface	Noah	Noah	Noah
Chemistry	Gas phase chemistry	CBM-Z	CBM-Z	CBM-Z
	Aerosol	MOSAIC	MOSAIC	MOSAIC
	Photolysis	Madronich F-TUV	Madronich F-TUV	Madronich F-TUV
	Aerosol feedback	Open	Open	Open

### Datasets and experimental configuration

The model was initialized with the initial meteorological and boundary conditions using the National Center for Environmental Prediction (NCEP) Final Analysis (FNL) reanalysis datasets with a spatial resolution of 1° × 1° and a 6-h temporal resolution. Community Atmosphere Model with chemistry (CAM-CHEM) data were adopted as the chemical conditions at the lateral boundaries. Meteorological and chemical observational datasets comprising historical air quality data recorded in China were used for the model evaluation. The evaluated meteorological variables included the temperature at 2 m, wind speed at 10 m, and wind direction at 10 m. The evaluated chemical species included hourly PM_2.5_ concentrations and hourly PM_10_ concentrations. For the emission inventory, we used the Multiresolution Emission Inventory for China (MEIC) data, which provided all anthropogenic emissions of eight species, including sulfur dioxide (SO_2_), nitrogen oxides (NO_X_), carbon monoxide (CO), nonmethane volatile organic compounds (NMVOCs), ammonia (NH_3_), organic carbon (OC), respirable particulate matter (PM_10_), and fine particulate matter (PM_2.5_), in China; these emissions species have been divided into five source departments: electric power, industry, civil use, transportation, and agriculture. This inventory has been widely used to address regional air quality modeling^60^.

The simulation period of this study covered from December 10 to 29 in 2019 and removed the 7-day model spin-up time. This study used December 17–29 for analysis. Meteorological conditions can be affected by aerosols, as indicated by Yang et al.^61^, therefore, the model was initialized with the same initial meteorological and boundary conditions. The model uses the same FNL data to provide initial meteorological and boundary conditions in different simulation schemes to ensure the consistency of meteorological conditions in different simulation experiments. Simulations were run separately for the five different emission-control scenarios, namely, S1, S2, S3, S4, and S5 (Table 2). The S1 scenario corresponded to the situation in which the emissions of pollution sources were considered in all regions in the study area (Zhengzhou and six cities around Zhengzhou). S2, S3, S4, and S5 corresponded to pollution control scenarios in Zhengzhou and in the areas to the northeast of Zhengzhou (Xinxiang and Kaifeng), to the northwest of Zhengzhou (Luoyang and Jiaozuo), and to the south of Zhengzhou (Pingdingshan and Xuchang), respectively. The contribution of each region was calculated by using the following formulas:

1 $${\mathrm{C}}_{\mathrm{x}}=\mathrm{C}-{\mathrm{C}}_{\mathrm{z}}$$

2 $${\mathrm{P}}_{\mathrm{x}}=\frac{{\mathrm{C}}_{\mathrm{x}}}{\mathrm{C}}\times 100\mathrm{\%}$$

where $\mathrm{C}$ represents the benchmark PM concentration; ${\mathrm{C}}_{\mathrm{z}}$ is the PM concentration when the emissions of the region are set to zero; ${\mathrm{C}}_{\mathrm{x}}$ represents the difference in PM concentrations between emissions that were turned on and off in the region; and ${\mathrm{P}}_{\mathrm{x}}$ represents the contribution of emissions from the region. Similar methods have been used in other air-quality-modeling studies^62,63^.

**Table 2:** Description of simulation scenarios.

Code	Emission-control scenarios
S1	Considering all pollution sources in the study area (Zhengzhou and six surrounding cities)
S2	Controlling the pollution sources in Zhengzhou
S3	Controlling the pollution sources in the northeast of Zhengzhou (Xinxiang and Kaifeng)
S4	Controlling the pollution sources in the northwest of Zhengzhou (Luoyang and Jiaozuo)
S5	Controlling the pollution sources in the south of Zhengzhou (Pingdingshan and Xuchang)

## Results

### Model performance evaluation

**Figure 2:**
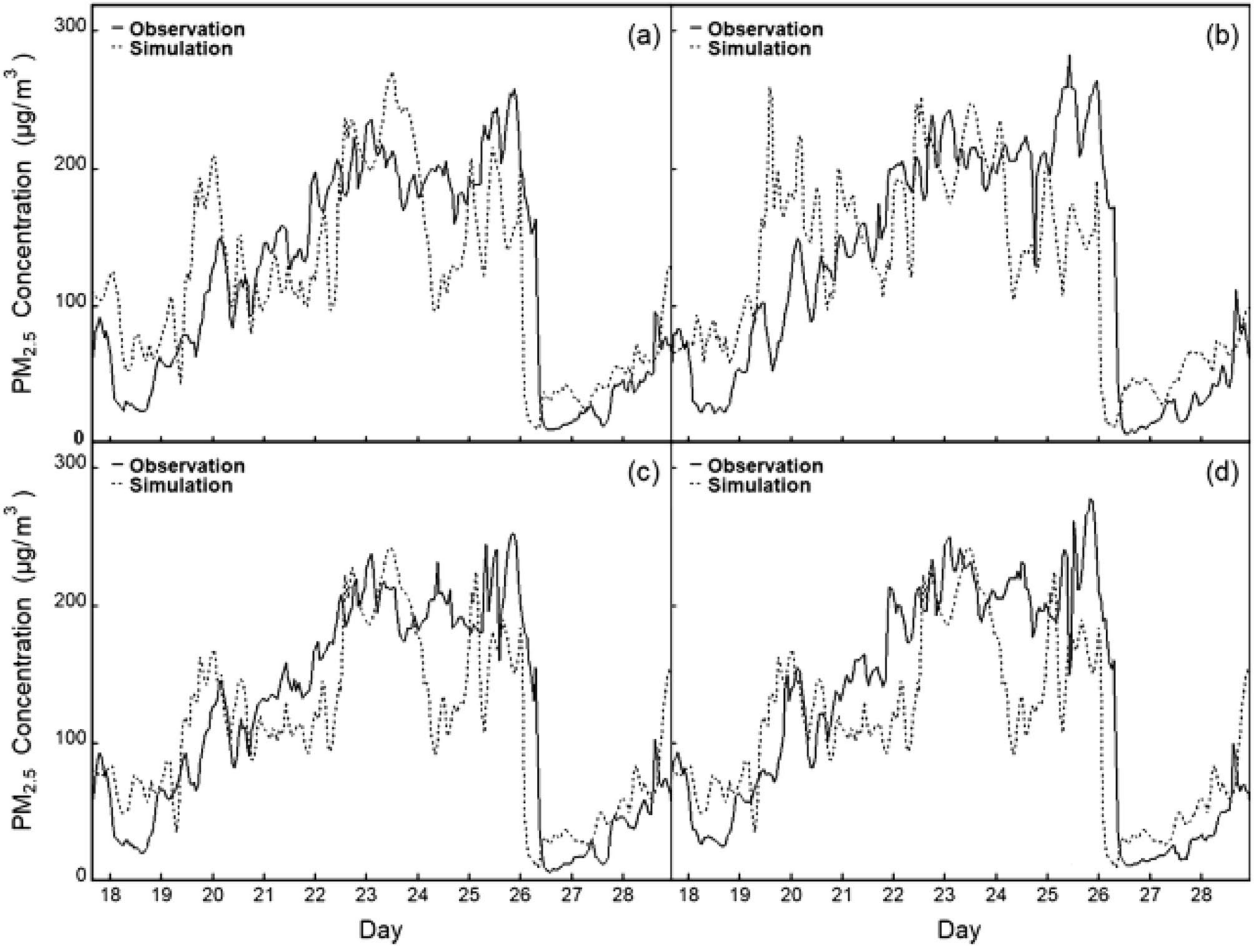
Hourly variations in observed (solid lines) and simulated (dashed lines) PM_2.5_ concentrations at four monitoring sites in Zhengzhou during December 18–29, 2020. (**a** is the verification of observed and simulated values at 1316A station; **b** is the verification of observed and simulated values at 1319A station; **c** is the verification of observed and simulated values at 1320A station; **d** is the verification of observed and simulated values at 1324A station).

**Figure 3:**
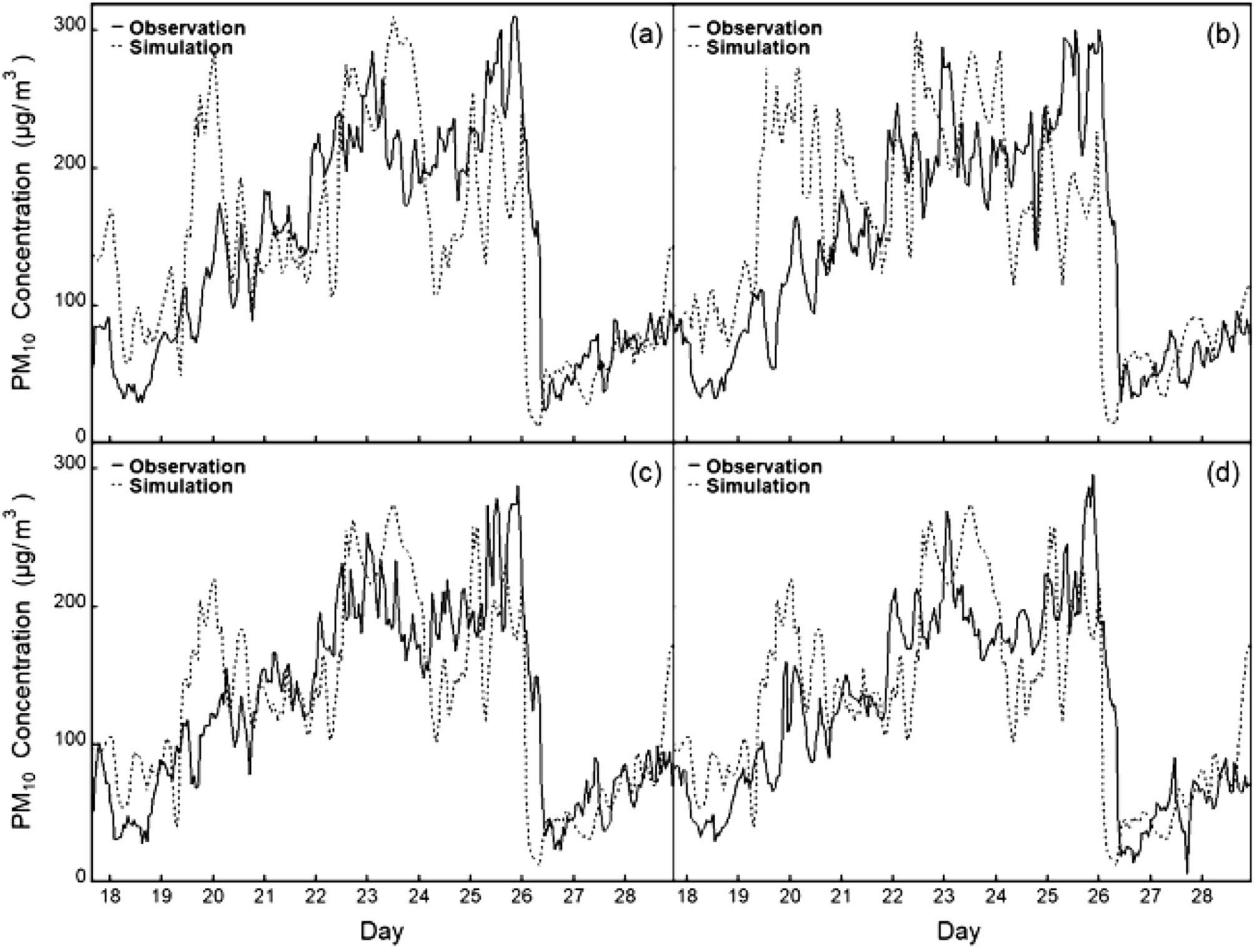
Hourly variations in observed (solid lines) and simulated (dashed lines) PM_10_ concentrations at four monitoring sites in Zhengzhou during the study period. (**a** is the verification of observed and simulated values at 1316A station; **b** is the verification of observed and simulated values at 1319A station; **c** is the verification of observed and simulated values at 1320A station; **d** is the verification of observed and simulated values at 1324A station).

To evaluate the performance of the WRF/Chem model, we compared the simulated and measured PM_2.5_ and PM_10_ concentrations (Figs. 2 and 3). The temporal trends of the simulated PM_2.5_ and PM_10_ concentrations were consistent with the observations at all 4 observation sites. In addition, these figures suggested the existence of temporal discrepancies between the peak simulated concentrations and peak observed concentrations. We can see that the WRF/Chem model can accurately simulate PM concentrations in Zhengzhou.

The validation results of the chemical and meteorological fields are shown in Table 3. The correlation coefficients (R) were 0.67, 0.55, 0.67, 0.38, 0.85 and 0.6 for the PM_2.5_ concentration, PM_10_ concentration, wind direction (DIR), wind speed (SPD), temperature (TMP) and precipitation (PRE), respectively. The highest R and lowest mean bias (MB) values were obtained for the simulated surface temperature. For the wind field simulation, the simulation deviation of the wind speed was - 0.78 m s^-1^, NMB was - 18%, and the correlation coefficient was approximately 0.4. The simulation deviation of the wind direction was - 6.15°, NMB was - 4%, and the correlation coefficient was 0.67. In general, the simulated wind speed was underestimated to a certain extent, which may have been caused by the wind field assimilation parameters of the four dimensional data assimilation (FDDA) used in this study for the WRF/Chem model. However, this was a slightly better estimation than those in other similar studies^52,64,65^. Overall, regardless of which site was selected, the simulated results agreed well with observations of atmospheric pollutants during the period investigated. The intercomparisons between simulated and observed concentrations indicated that WRF/Chem notably reproduced the observed time series of PM_2.5_ and PM_10_. However, the model tended to overestimate the concentrations of PM_2.5_ and PM_10_.

**Table 3:** Performance in meteorological conditions and PM concentrations of the WRF/Chem model in Zhengzhou.

	Obs	Sim	MB	NMB	NME	RMSE	R
PM_2.5_ (μg m^−3^)	102.97	122.04	19.07	0.19	0.45	57.79	0.67
PM_10_ (μg m^−3^)	119.10	148.47	29.36	0.25	0.47	71.76	0.55
DIR (°)	165.37	159.22	- 6.15	- 0.04	0.30	95.19	0.67
SPD (m s^−1^)	4.44	3.66	- 0.78	- 0.18	0.49	2.71	0.38
TMP (°C)	4.43	5.19	0.76	0.17	0.44	2.34	0.85
PRE (mm)	0.03	0	- 0.03	- 0.93	0.94	0.12	0.6

### Spatial and temporal variations in PM concentrations

**Figure 4:**
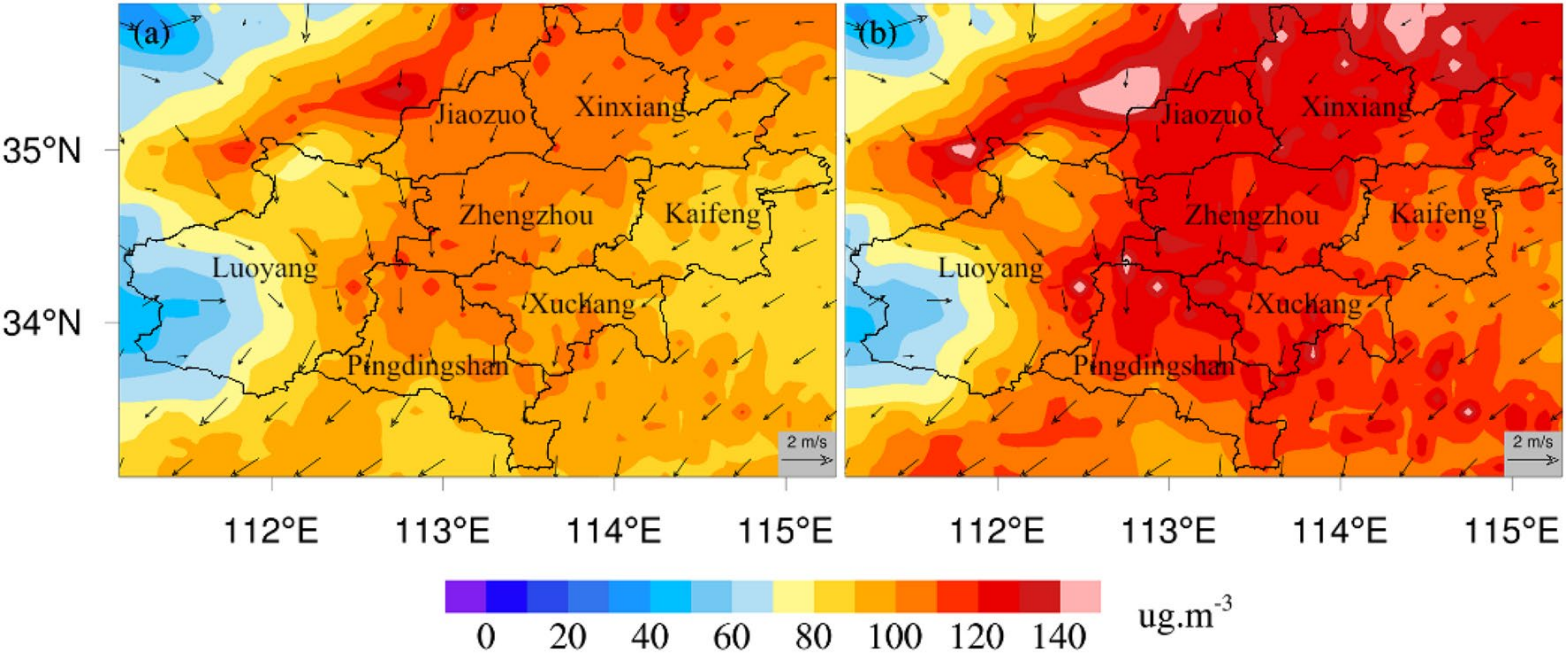
Spatial distributions of PM_2.5_ (**a**) and PM_10_ (**b**) monthly mean concentrations in Zhengzhou.

The spatial distribution of the mean PM_2.5_ concentrations was basically consistent with that of the mean PM_10_ concentrations (Fig. 4). However, the PM_2.5_ concentrations were approximately 10–20 μg m^-3^ lower than the PM_10_ concentrations at the same location. The maximum simulated PM_2.5_ concentration was obtained in the northwest area of Jiaozuo (Fig. 4a). This might have been related to the absence of obvious organized wind directions and weak wind speeds in this region. In most parts of Zhengzhou, the PM_2.5_ concentrations exceeded 100 μg m^-3^, characterizing high-PM_2.5_-concentration environments. The lowest PM_2.5_ concentration in Zhengzhou was over 80 μg m^-3^. In this area, the wind was strong, and the wind direction was mainly southward. High PM_2.5_ concentrations were distributed in a band in this area. For PM_10_, a similar spatial distribution was found; the highest PM_10_ concentration was found in the northwest area of Jiaozuo and was above 140 μg m^-3^, while Zhengzhou mostly had PM_10_ concentrations over 100 μg m^-3^. Zhengzhou and the area north of Zhengzhou were mainly affected by north winds. The high PM pollution concentrations identified in this area extended slightly from north to south.

**Figure 5:**
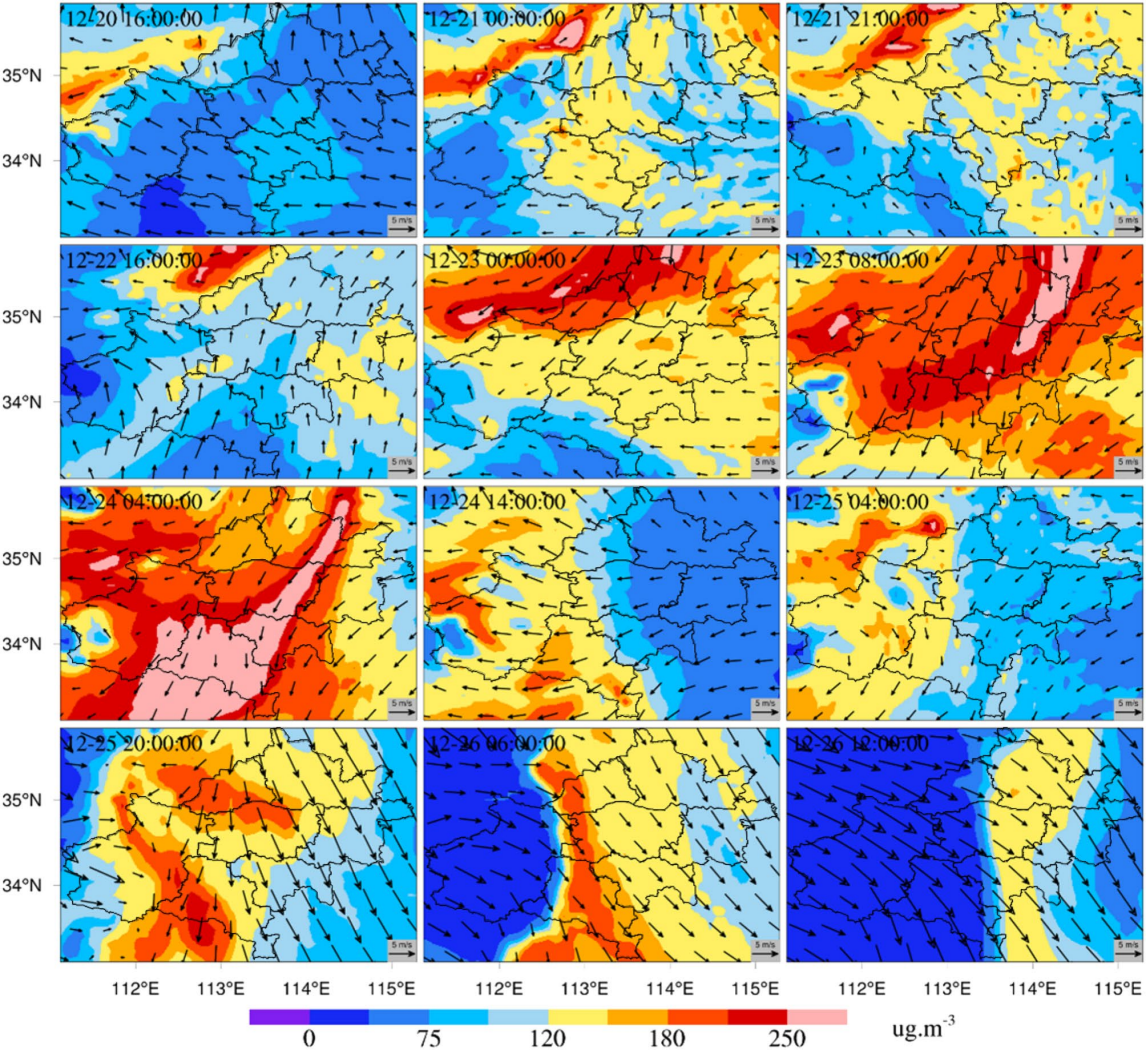
Spatial and temporal process of PM_2.5_ concentrations in this pollution episode.

Figure 5 shows the temporal variation in the simulated PM_2.5_ concentration in Zhengzhou under S1. At 16:00 on December 20, the PM_2.5_ concentrations were between 35 and 100 μg m^-3^ in most parts of Zhengzhou and its surrounding areas. At this time, the southeast wind speed reached 5 m s^-1^. From 00:00 to 21:00 on December 21, pollutants accumulated in Zhengzhou with weak wind speeds. The highest PM_2.5_ concentration was more than 120 μg m^-3^. At 16:00 on December 22, a slight increase appeared in the wind speed, and the wind turned southerly. The accumulated pollutants were blown away by winds. From 16:00 on December 20 to 16:00 on December 22, a high-PM_2.5_-concentration region was located north of Jiaozuo and Luoyang. At 08:00 on December 23, the PM_2.5_ concentration in Zhengzhou was above 180 μg m^-3^ and even reached levels of 250 μg m^-3^ and above in northeastern Zhengzhou. The PM_2.5_ concentrations were between 160 and 220 μg m^-3^ in the surrounding areas of Zhengzhou. At this moment, the northeast was the prevailing wind direction, and the wind speed was high. By 04:00 on December 24, the wind weakened, and the main wind direction became consistent with that recorded before. The heavily polluted area gradually moved southward. At 14:00 on December 24, however, the wind direction changed greatly and became an easterly wind. The pollutants in Xinxiang, Kaifeng, Xuchang and east of Zhengzhou were eradicated. The PM_2.5_ concentrations dropped to below 75 μg m^-3^. At 04:00 on December 25, the wind weakened further, and the wind direction became disorganized. At 20:00 on December 25, the wind speeds in Xinxiang, Kaifeng, Xuchang and east of Zhengzhou increased, and the wind direction shifted to the northwest. PM_2.5_ pollution started to spread rapidly to the east. At 06:00 on December 26, the main wind direction changed to a westerly wind, and the wind speed clearly increased in the northwest region. The high-pollution area moved eastward, and the PM_2.5_ concentrations in Zhengzhou slightly decreased. At 12:00 on December 26, the wind speeds increased significantly, and the main wind direction remained northwest. The PM_2.5_ concentration clearly decreased to below 120 μg m^-3^ in most areas.

**Figure 6:**
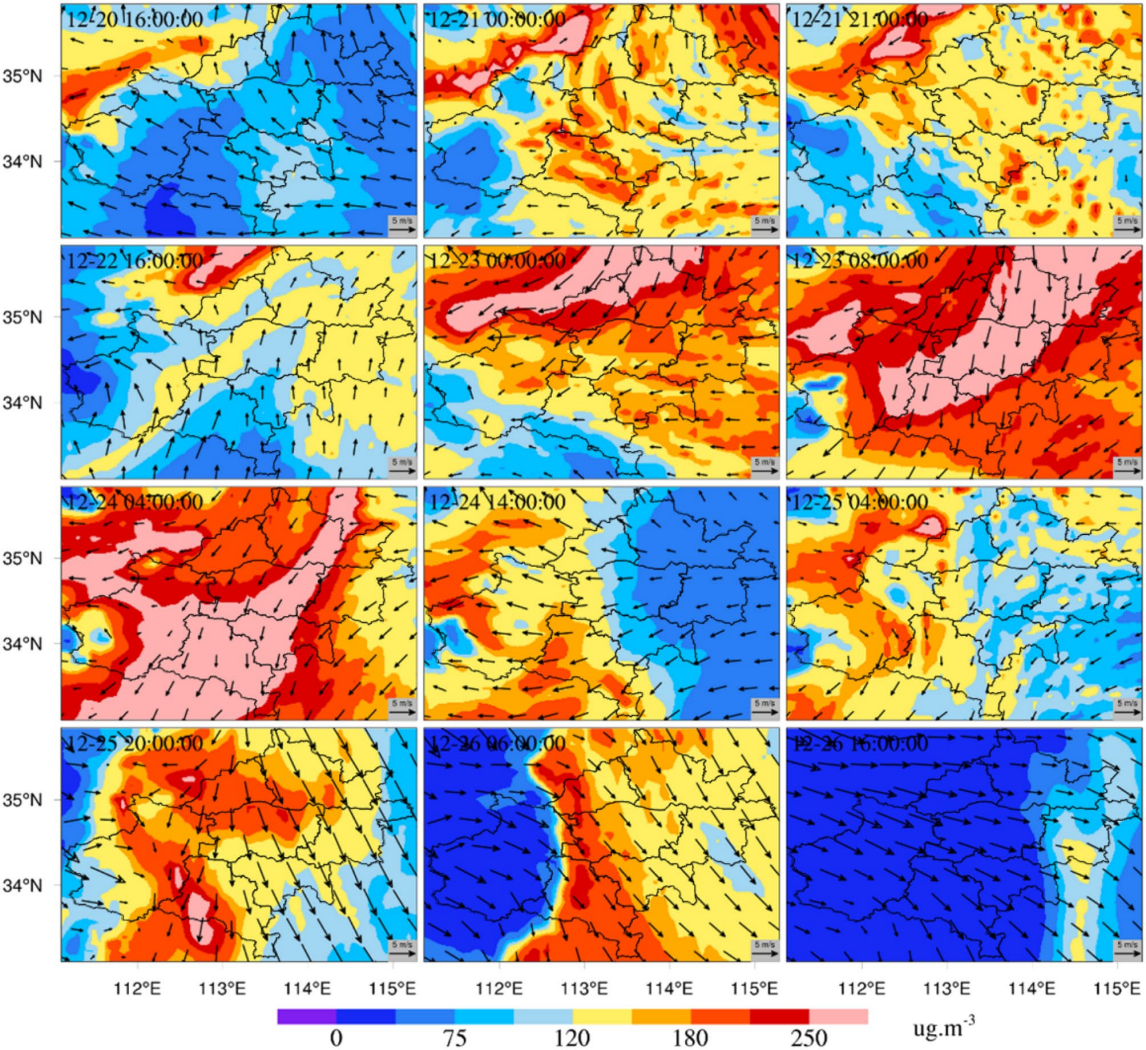
Spatial and temporal process of PM_10_ concentrations in this pollution episode.

Similar temporal variations in the simulated PM_10_ concentrations occurred in the simulated area (Fig. 6). At 16:00 on December 20, the spatial distribution of PM_10_ concentrations was similar to that of PM_2.5_ concentrations. On December 21, the high-PM_10_-concentration situation lasted all day. At 16:00 on December 22, an organized wind direction emerged. Due to the south winds, the accumulated pollutant concentrations were reduced in Zhengzhou. At 00:00 on December 23, an obvious northerly wind could be identified north of Zhengzhou, and the high pollutant concentrations in this area spread to the south. At 08:00 on December 23, the PM_10_ concentrations were above 250 μg m^-3^ in Zhengzhou. The northeast winds caused high pollutant concentrations to continuously spread southward. At 04:00 on December 24, the high-pollutant-concentration area changed from impacting Zhengzhou and Xinxiang to spanning Luoyang, Pingdingshan and Xuchang. At 14:00 on December 24, the main wind direction became easterly, and high pollutant concentrations were thus transferred westward. Then, the wind conditions began to weaken and became disordered. At 04:00 on December 25, a slight PM_10_ accumulation occurred east of Zhengzhou. At 20:00 on December 25, the PM_10_ concentration was higher than that recorded hours earlier. At 06:00 on December 26, however, the winds in Zhengzhou and its surrounding areas were organized and developed into northwest winds with high speeds. Several hours of northwesterly winds caused the PM_10_ concentrations in Zhengzhou and its surrounding areas to significantly and rapidly decrease. At 16:00 on December 26, the PM_10_ concentrations were below 75 μg m^-3^ in most of Zhengzhou.

### Trajectory analysis of the continuous heavy pollution process

**Figure 7:**
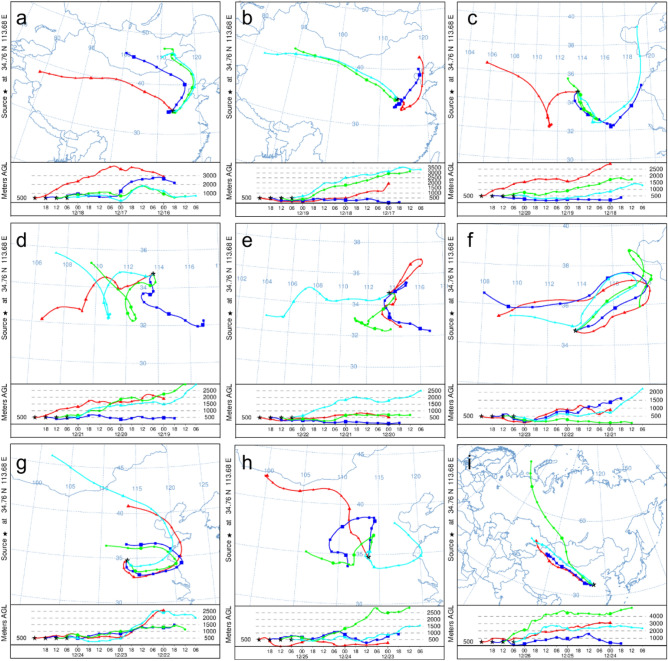
Backward trajectory from December 19 to 27, 2019 (**a**–**i** represent December 19, December 20, December 21, December 22, December 23, December 24, December 25, December 26 and December 27; the light blue track, green track, dark blue track, and red track indicate the air mass track arriving at 00:00, 06:00, 12:00 and 18:00, respectively).

In Fig. 7, the 72-h backward trajectories of the air mass arriving at Zhengzhou sampling point (1016A) at 00:00, 06:00, 12:00 and 18:00 every day were simulated for 9 consecutive days (December 19–27, 2019). The starting point of the simulated air mass was a height of 500 m above the Zhengzhou sampling point. On Dec. 19, this air mass was mainly a long-distance one from the north (Fig. 7a). On Dec. 20, the air mass was still dominated by long-distance air mass from the northwest (Fig. 7b). On Dec. 21, the air mass changed, the long-distance air mass from the northwest gradually disappeared, and the short-distance air mass from the east and southeast to Zhengzhou increased (Fig. 7c). On Dec. 22, the air mass changed again, as the air mass from the northeast disappeared. It comprised mainly a high-altitude air mass from the west (> 1500 m) and a small amount of low-altitude air mass from the southeast (< 500 m) (Fig. 7d). On Dec. 23, the air mass changed significantly, adding a short-range low-level air mass (< 1000 m) from the south and north, and the concentration of PM increased significantly (Figs. 5, 6, 7e). On Dec. 24, the low-altitude air mass from close range decreased, and the high-altitude air mass (> 1500 m) from the northwest increased. The PM concentration decreased (Figs. 5, 6, 7f). On Dec. 25, the long-distance high air mass (> 1500 m) from the north was the main air mass source, and the short-distance air mass source had a height of more than 1000 m (Fig. 7g). On Dec. 26, the low-level air mass (< 500 m) from the north began to enter, and the particulate concentration increased (Figs. 5, 6, 7h). On Dec. 27, a long-distance high air mass (> 2000 m) from the northwest became the primary source, and the particulate concentration at the observation station showed a significant downward trend (Fig. 7i). By analyzing the reverse trajectory of air mass, we found that in the heavy pollutant episode, an air mass less than 1000 m can cause PM to accumulate. The air mass above 1000 m can reduce the PM concentration, so the inflow of a clean air mass at a high altitude can effectively alleviate the PM pollution.

### Effects of local and surrounding emissions

**Figure 8:**
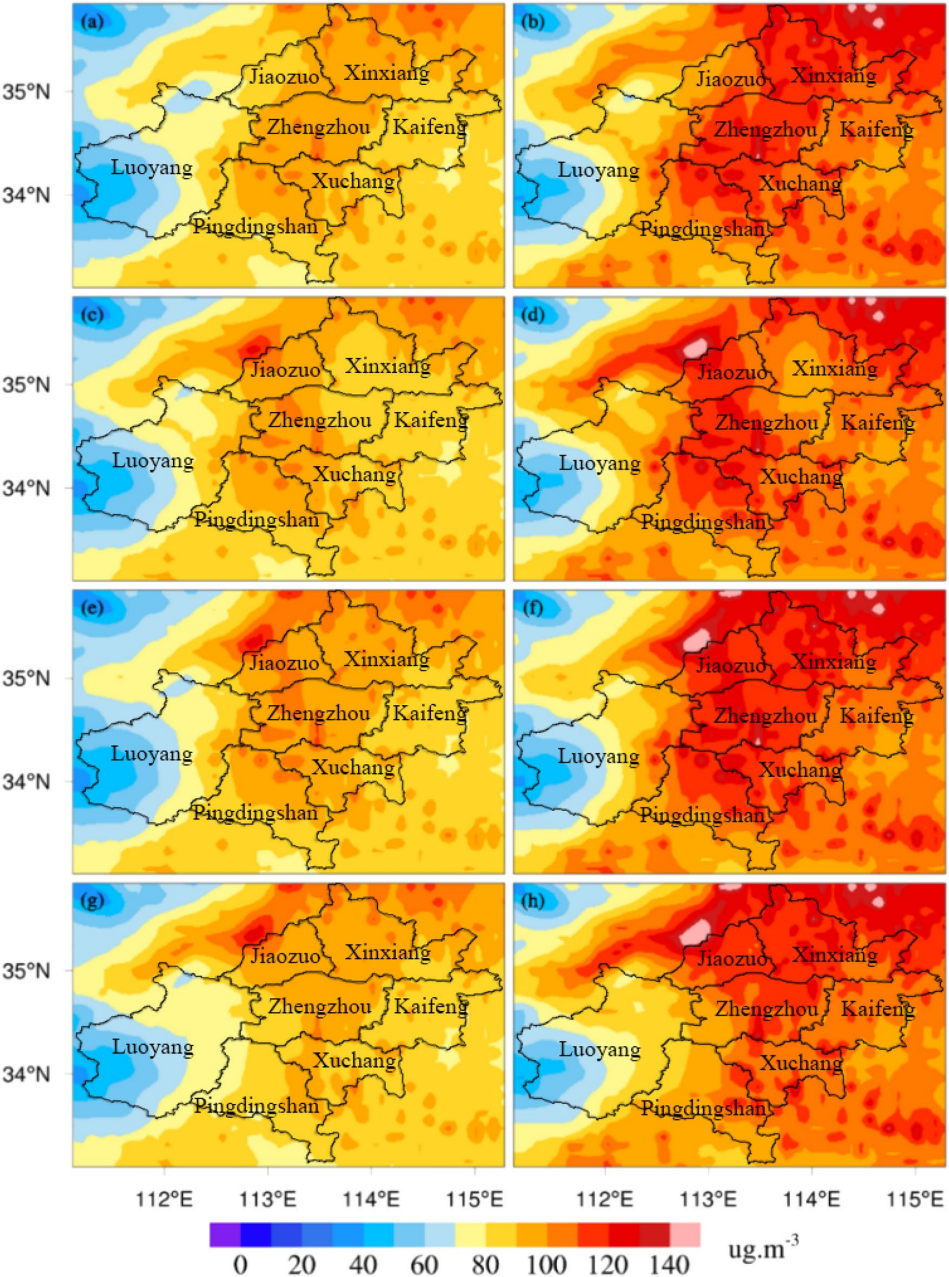
Spatial distributions of PM monthly mean concentrations under different scenarios (PM_2.5_: **a**,**c**,**e**,**g** correspond to scenarios S2, S3, S4, and S5, respectively; PM_10_: **b**,**d**,**f**,**h** correspond to scenarios S2, S3, S4, S5, respectively).

There were different spatial distributions of the simulated mean PM_2.5_ and PM_10_ concentrations under different schemes (Fig. 8). Decreased PM_2.5_ and PM_10_ concentrations were observed in Zhengzhou and its surrounding areas under S2, S3, S4 and S5. Under scheme S2, the pollution sources in Zhengzhou were controlled. There was a significant drop in PM_2.5_ concentrations in the region northwest of Zhengzhou (Fig. 8a). The mean simulated PM_2.5_ concentration in Zhengzhou decreased by approximately 10 μg m^-3^. A similar decrease in PM_10_ concentrations in northwestern Zhengzhou can be seen in Fig. 8b. The emissions of Zhengzhou impacted the surrounding areas, and the largest impact occurred in the area to the northwest of Zhengzhou. Under scheme S3, the pollution sources in the area to the northeast of Zhengzhou (Xinxiang and Kaifeng) were controlled. The simulated PM_2.5_ concentrations (Fig. 8c) were highest in the region to northwest of Zhengzhou but were slightly lower than those displayed in Fig. 4a. The PM_2.5_ concentrations simulated at the junction of Zhengzhou, Xinxiang and Kaifeng were lower than those obtained in the base simulation. The pollution conditions in this area were more sensitive to the impact of emissions under scheme S3 than in other areas. The PM_10_ concentrations simulated under scheme S3 showed a similar change (Fig. 8d). Under scheme S4, the pollution sources in the area to the northwest of Zhengzhou (Luoyang and Jiaozuo) were controlled. the changes in the simulated spatial distributions of PM_2.5_ and PM_10_ concentrations were the least obvious among the experiments comprising different schemes, and the pollutant concentrations decreased slightly (Fig. 8e,f). Under scheme S5, the pollution sources in the area to the south of Zhengzhou (Pingdingshan and Xuchang) were controlled. The simulated PM_2.5_ and PM_10_ concentrations in regions to the south and southwest of Zhengzhou decreased significantly (Fig. 8g,h). The PM_10_ concentrations obviously decreased in western Zhengzhou. Among the four sensitivity experiments, the simulated change in the concentration of pollutants was the largest under scheme S5 compared to the case of scheme S1. Emissions from Zhengzhou significantly impacted the pollutant concentrations in the region to northwest of Zhengzhou. The impacts of emissions from Xinxiang and Kaifeng were obvious at the junction of Zhengzhou, Xinxiang and Kaifeng. In the area northwest of Zhengzhou, the impacts of local emissions were more obvious than those in other regions. Emissions from the southern region obviously impacted the southern and western areas of Zhengzhou.

**Figure 9:**
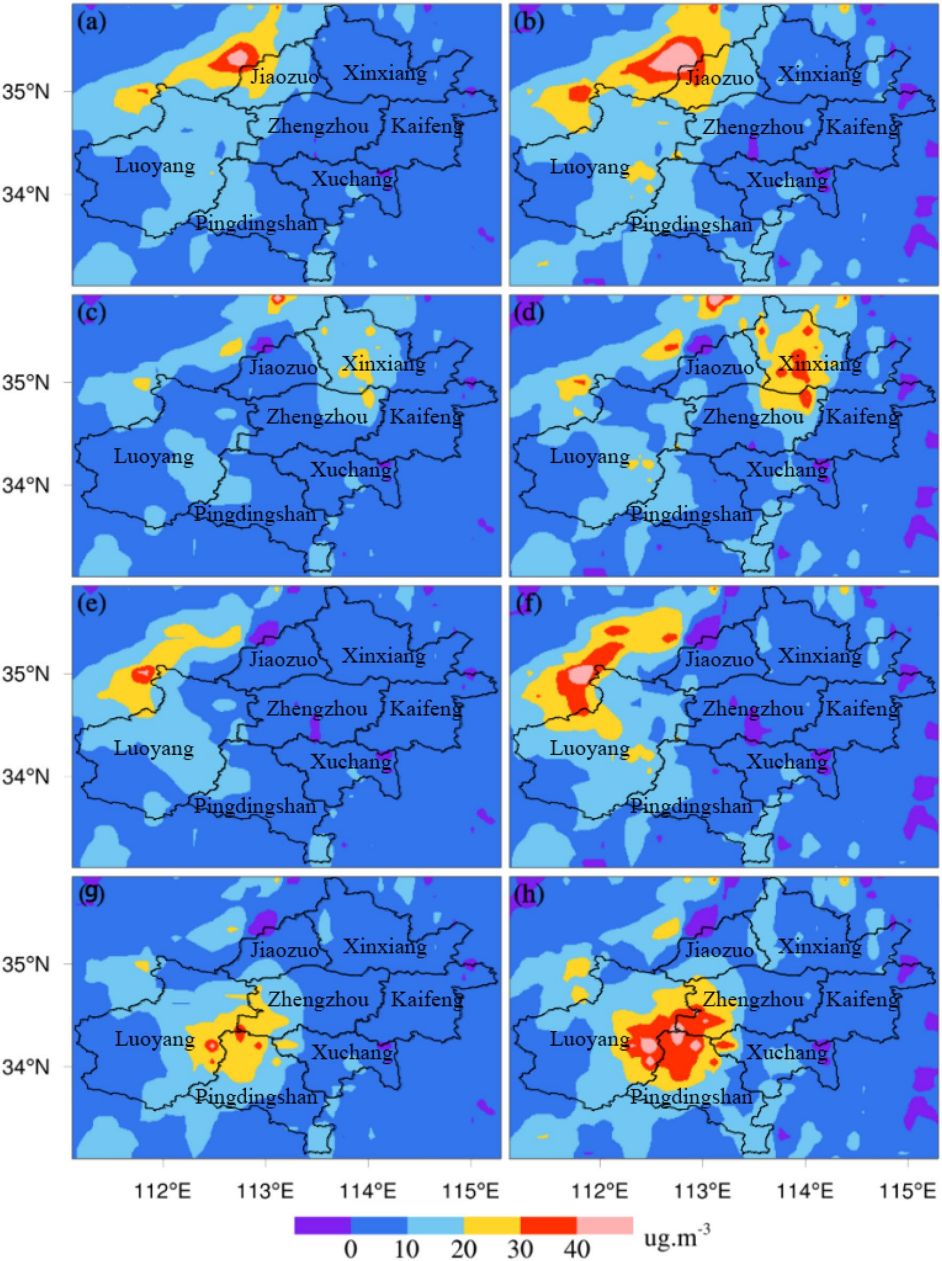
Spatial differences in PM concentrations between control scenarios (PM_2.5_: **a**,**c**,**e**,**g** denote scenarios S2, S3, S4, and S5, respectively; PM_10_: **b**,**d**,**f**,**h** denote scenarios S2, S3, S4, and S5, respectively).

Figure 9 shows the spatial distribution of the simulated values under the S1 scheme minus the simulated values under the control scenarios. As described in the previous section, the pollutant concentrations obviously decreased after removing local pollutant emissions from Zhengzhou (Fig. 9a,b). The area with the largest reduction in pollutant concentrations was located in the region northwest of Zhengzhou, with differences above 40 μg m^-3^. The concentration of pollutants changed dramatically in an area of northeastern Zhengzhou under S3 (Fig. 9c,d). The reductions in PM_2.5_ concentrations were approximately 20 μg m^-3^, and the reductions in PM_10_ concentrations were even higher than 30 μg m^-3^ in northeastern Zhengzhou. The emissions from Xinxiang and Kaifeng had obvious impacts on the reduced pollutant concentrations in northeastern Zhengzhou. Under S5, the reductions in PM_2.5_ and PM_10_ concentrations were approximately 40 μg m^-3^ at the junction of Zhengzhou, Luoyang and Pingdingshan. As in the other experimental schemes, the effect of emission-control measures on PM_10_ concentrations was greater than that on PM_2.5_ concentrations. The experiments represented by S2 and S4 had little influence on reducing PM_2.5_ and PM_10_ concentrations in Zhengzhou. In general, emissions from the pollution sources in Zhengzhou had a great impact on the particulate matter concentration in the area to the northwest of Zhengzhou. In addition, the emission of pollution sources in the area to the south of Zhengzhou had a more obvious impact on the area to the northwest of Zhengzhou.

**Figure 10:**
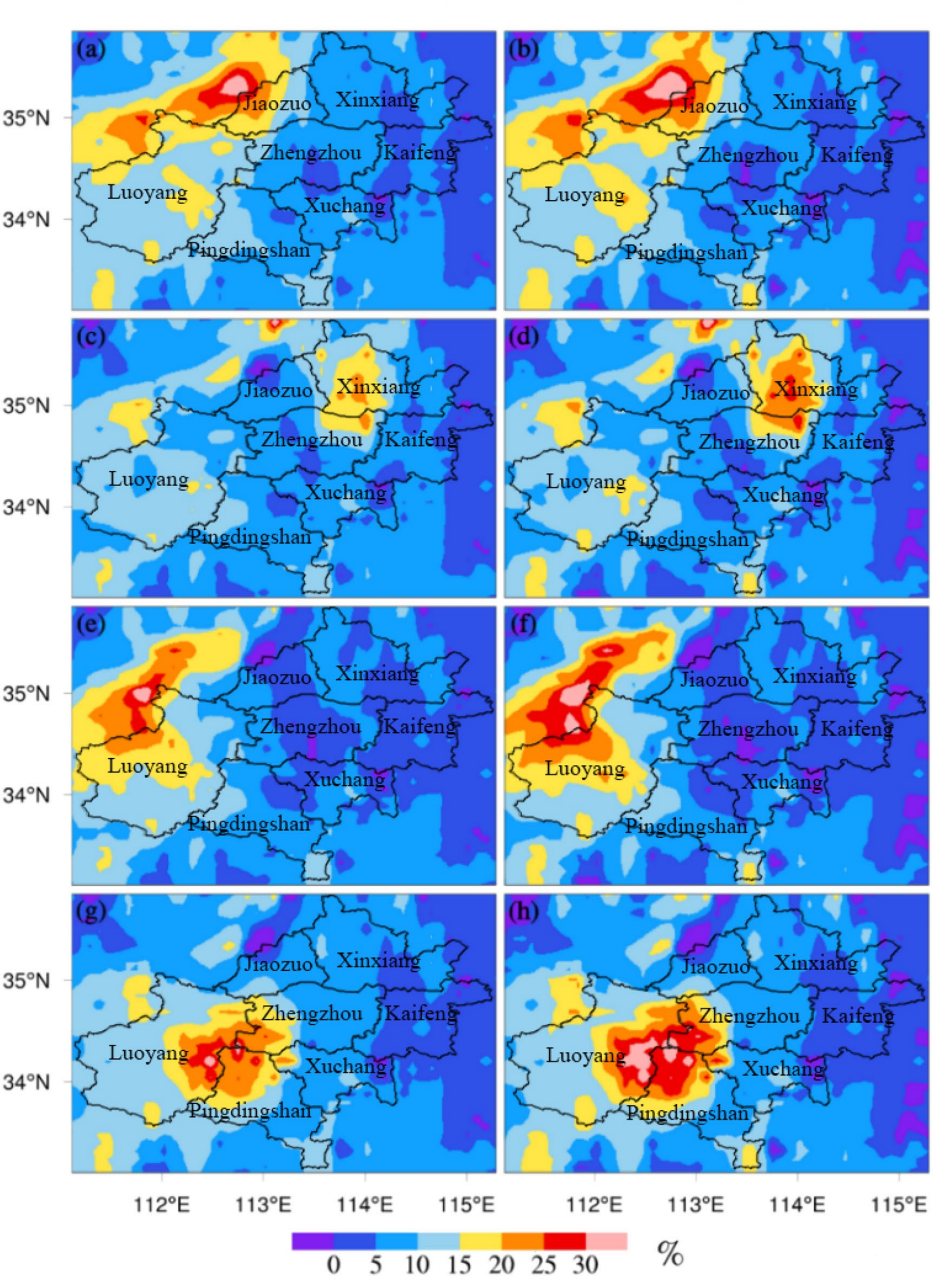
Spatial distributions of the contribution rates of different control scenarios to the PM concentrations in Zhengzhou (PM_2.5_: **a**,**c**,**e**,**g** denote scenarios S2, S3, S4, and S5, respectively; PM_10_: **b**,**d**,**f**,**h** denote scenarios S2, S3, S4, and S5, respectively).

The formula mentioned in section "Model settings" was used to calculate the contribution rates of emissions to air pollution concentrations in different regions. The local emissions of Zhengzhou had a certain contribution to its local pollutant concentrations, while the contribution to the central area of Zhengzhou was relatively weak (Fig. 10a,b). The contribution to the area to the northwest of Zhengzhou (the junction of Luoyang and Jiaozuo) peaked at more than 20%. The emissions from the area to the northeast of Zhengzhou strongly contributed to the PM_2.5_ concentrations in the northeast area in Zhengzhou (Fig. 10c). The contribution to the PM_10_ concentrations in the northeast area in Zhengzhou was relatively strong, at more than 25% (Fig. 10d). The contribution to the PM_2.5_ and PM_10_ concentrations were the highest in the northeast of Zhengzhou, gradually decreasing to the south, and the lowest in the downtown area. The emissions from the area to northeast Zhengzhou had an impact on northeast Zhengzhou, and the impact on the urban center was relatively limited. The emissions from the area to northwest of Zhengzhou had little influence on the local pollutant concentration in Zhengzhou (Fig. 10e,f). The contribution was approximately 5%. The contribution of the emissions from the area to northwest Zhengzhou to the center of Zhengzhou was negative. The contribution of these emissions to the junction of Luoyang and Jiyuan peaked at more than 30%. The emissions from the area to the south of Zhengzhou strongly impacted the local pollutant concentrations in Zhengzhou. The contribution of these emissions to the local pollutant concentration in Zhengzhou was above 5%, and this contribution was even above 25% southwest of Zhengzhou (Fig. 10g). The contribution distribution shown in Fig. 10h is similar to that shown in Fig. 10g; the contributions to PM_10_ concentrations in southwestern Zhengzhou were above 30%. The contribution of the emissions from the area to the south of Zhengzhou (Pingdingshan and Xuchang) to PM_2.5_ and PM_10_ concentrations in Zhengzhou was larger than that in other areas (the area to the northeast and northwest of Zhengzhou). The contribution of these emissions to PM_2.5_ and PM_10_ concentrations at the junction of Luoyang, Pingdingshan and Zhengzhou peaked at more than 35%.

**Figure 11:**
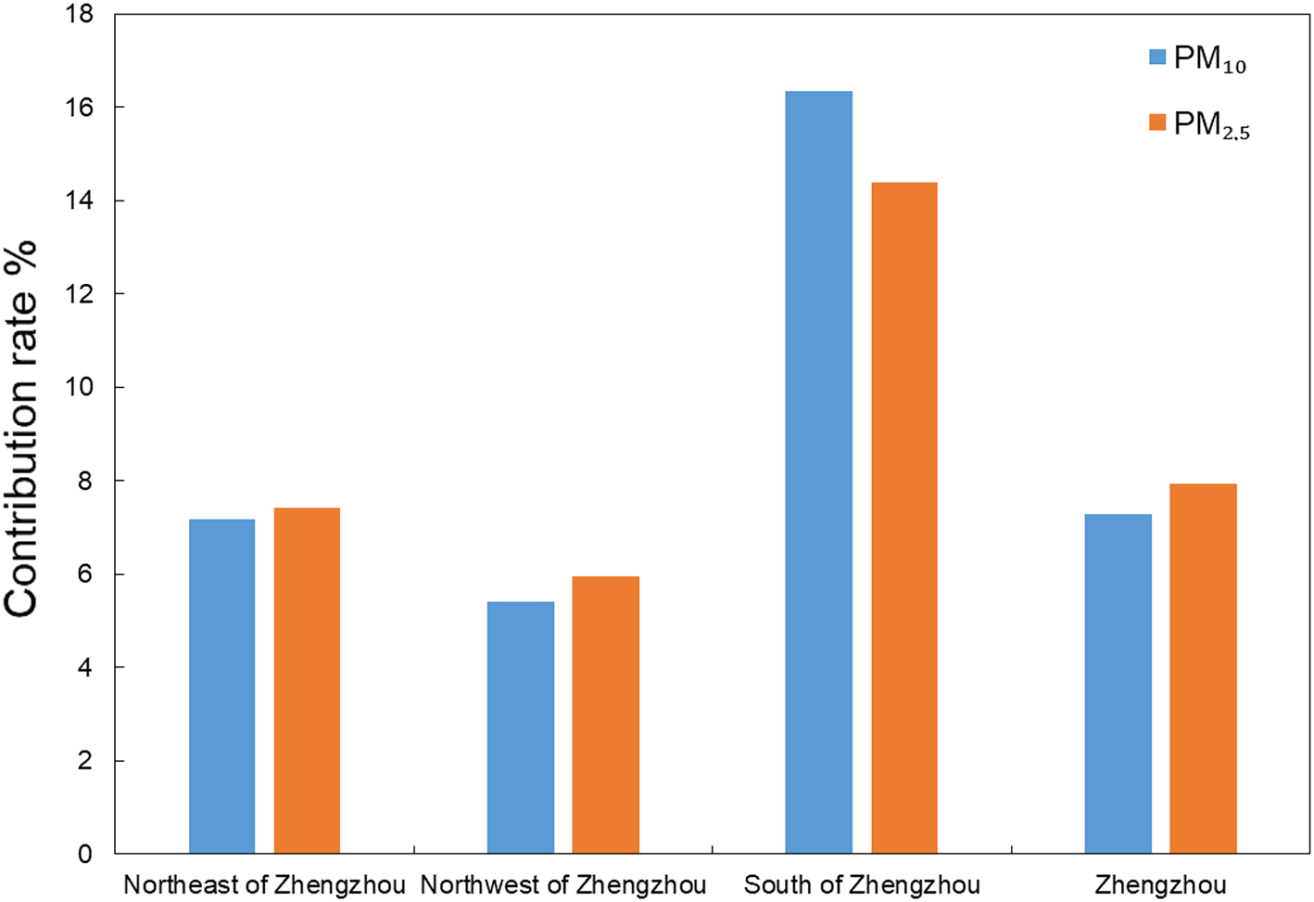
Contribution rate of anthropogenic emissions over surrounding areas to PM concentration in Zhengzhou.

The emissions from the region to the south of Zhengzhou (Pingdingshan and Xuchang) had the most serious impacts on the pollutant concentrations in Zhengzhou (Fig. 11). The contribution of the emissions from this area to the PM_2.5_ concentrations in Zhengzhou was 14.39%. The contribution of the emissions from this area to the PM_10_ concentrations in Zhengzhou was 16.34%, 1.95% higher than that to the PM_2.5_ concentrations. The emissions from the region to northwest Zhengzhou (Luoyang and Jiaozuo) had the weakest impact on pollutant concentrations in Zhengzhou. However, the contribution of the emissions from this area to PM_10_ concentrations (5.40%) was lower than that to PM_2.5_ concentrations (5.96%). The emissions from the area to northeast of Zhengzhou (Xinxiang and Kaifeng) and the local area of Zhengzhou had similar impacts on the pollutant concentrations in Zhengzhou. The contributions of emissions from the area to the northeast of Zhengzhou and the local area of Zhengzhou to the PM_10_ concentrations in Zhengzhou were 7.18% and 7.29%, respectively. For the PM_2.5_ concentrations, the contributions of emissions from the area to the northeast of Zhengzhou and the local area of Zhengzhou were 7.42% and 7.94%, respectively.

## Discussion

This study proved that the localized WRF/Chem model can effectively simulate regional-scale particulate pollution. The results show that by using the verification methods implemented in previous studies to verify the simulated peak concentrations of PM_2.5_ and PM_10_ and the temporal variations in particulate concentration, wind speed, wind direction and temperature, the WRF/Chem model demonstrates high reliability for simulating particulate pollution temporal and spatial change patterns in Zhengzhou and its surrounding areas^66,67^. The temporal and spatial sequence diagram of simulated and observed particulate concentrations from December 10–29, 2019, showed that the concentrations of PM_2.5_ and PM_10_ calculated by WRF/Chem were consistent with the daily variation trend of observed data, indicating that WRF/Chem provides a relatively reasonable estimate for the emission of particulate pollution. Overall, the NMB and R values are within the range of previous research results^68,69^.

The simulation results were highly similar to the particulate pollution pattern of Zhengzhou in winter simulated by Wang et al.^70^ and Hu et al.^71^. During this period, northeast winds prevailed in Zhengzhou, which greatly promoted the transportation of particulate pollutants from northeast to south. The maximum simulated concentrations of PM_2.5_ and PM_10_ in the Jiaozuo area were related to the lack of obvious organized wind direction and weak wind speed. Previous studies have proven that the pollution transmission process of particulate matter is greatly affected by meteorological factors. Wei et al. found that from October 8–11, 2014, the air quality in Beijing and Shijiazhuang continued to be heavily polluted over four days. On October 12, the weather situation changed, and systematic northerly winds developed in Northeast China, North China, Central China, East China and South China, with strong winds reaching 10 m/s in some parts, effectively removing pollutants. On the 12th, the air quality in Beijing, Shijiazhuang and other cities became excellent, and the continuous heavy pollution weather was effectively alleviated^72^. Strong wind is conducive to removing air pollutants in local areas and transporting air pollutants downwind^61^. The environmental conditions of continuous low wind speed are the main meteorological factors causing continuous pollution^73,74^. To further study the regional particulate matter transport in Zhengzhou and its surrounding areas, we investigated the hourly variation in simulated concentrations of PM_2.5_ and PM_10_ in Zhengzhou and its surrounding areas from December 20–26, 2019 (Figs. 5, 6). Driven by the northeast wind, the peak concentrations of surface PM_2.5_ and PM_10_ pushed southward from Xinxiang and Jiaozuo to Zhengzhou. In the early morning of December 24, 2019, the peak concentration of particulate matter pushed southward from Zhengzhou to Pingdingshan and Xuchang, which showed that regional PM migration had a significant impact on PM pollution.

Based on the effective reproduction of the pollution characteristics of PM_2.5_ and PM_10_ by the WRF/Chem model, sensitivity simulation experiments of different emission-control schemes were carried out to determine the impact of local and regional emissions on air quality in Zhengzhou. The simulation results under different emission-control schemes (Figs. 4, 7, 8) showed that in the four sensitivity tests, the change in simulated concentrations of PM_2.5_ and PM_10_ under scheme S5 was the largest compared with scheme S1. The local pollution discharge in Zhengzhou had an impact on the particulate pollution in the surrounding areas. The greatest impact was in the area to the northwest of Zhengzhou, where the impact was more obvious than in other areas. The spatial distribution changes in PM_2.5_ and PM_10_ concentrations simulated under scheme S4 were not significant in the tests with different schemes, and the emissions from the area to the northeast of Zhengzhou had no significant impact on any specific region. Emissions from the area to the south of Zhengzhou (Pingdingshan and Xuchang) significantly affected the southern and western regions of Zhengzhou.

According to the quantitative analysis of the simulation results, we found that the emissions from the area to south of Zhengzhou (Pingdingshan and Xuchang) had the most significant impact on the concentration of particulate pollutants in Zhengzhou. The contribution rate of emissions from this area to the PM_2.5_ concentration in Zhengzhou was 14.39%, and the contribution rate to the PM_10_ concentration was 16.34%. The emissions from the area to northwest Zhengzhou (Luoyang and Jiaozuo) had the weakest impact on the pollutant concentration in Zhengzhou. The contribution rates of emissions to PM_10_ and PM_2.5_ concentrations in this area were 5.40% and 5.96%, respectively. The surrounding areas contributed to the particulate pollution in Zhengzhou. According to the results, the contribution of local and surrounding emissions from pollution sources to the concentration of particulate matter in Zhengzhou was in the following order: emissions of the area to the south of Zhengzhou were greater than emissions of the local area; emissions of the local area were greater than emissions of the area to northeast of Zhengzhou; emissions of the area to the northeast of Zhengzhou were greater than emissions of area to the northwest of Zhengzhou. The local emission of Zhengzhou was also an important contributor to the particulate pollution in Zhengzhou, but it was not the only source of the particulate pollution in Zhengzhou. Pollution transport from the surrounding areas was an important source of particulate pollution in Zhengzhou. In addition, the emissions of neighboring provinces (such as Shanxi, Shaanxi, Anhui and Shandong) were also important contributors to particulate pollution in Zhengzhou^33^.

The objective of this study was to identify the contribution of anthropogenic emissions in different areas within Henan to the PM concentrations of Zhengzhou. Therefore, we mainly focused on quantifying the contribution of local and surrounding anthropogenic emissions to the PM concentration in Zhengzhou. However, this study did not simulate the contribution of different sectors (i.e., industry, transportation) to the PM concentration, which is important for proposing scientific emission reduction measures and also comprises the work that we will conduct next.

## Conclusions

This study simulated the spatial and temporal variations in PM concentrations by using the WRF/Chem model and quantified its contribution rates from local and neighboring regions of Zhengzhou during a severe PM pollution episode. Emissions from the area to the south of Zhengzhou (Pingdingshan and Xuchang) were the most important contributors to particulate pollution in Zhengzhou among all the cities surrounding Zhengzhou during this episode. This region contributed 14.4% to the PM_2.5_ concentration and 16.3% to the PM_10_ concentration in Zhengzhou. The local emissions and the emissions from the area to northeast Zhengzhou (Xinxiang and Kaifeng) had similar contributions to the PM_2.5_ (7.9% and 7.4%, respectively) and PM_10_ (7.3% and 7.2%, respectively) concentrations in Zhengzhou. The emissions from the area to the northwest of Zhengzhou (Luoyang and Jiaozuo) had the weakest contribution. The contributions of the emissions from this area to the PM_10_ and PM_2.5_ concentrations were 5.4% and 6.0%, respectively. We also found that the neighboring cities accounted for approximately 35% of the PM concentration in Zhengzhou, and approximately 2/3 of the contribution was transported from other regions, indicating that urban particulate pollution control to improve the urban air quality may be more effectively achieved by joint prevention and control in a wider area.

## Data Availability

The datasets generated during the current study are available from the corresponding authors on reasonable request.
